# Modelling of Breath and Various Blood Volatilomic Profiles—Implications for Breath Volatile Analysis

**DOI:** 10.3390/molecules27082381

**Published:** 2022-04-07

**Authors:** Paweł Mochalski, Julian King, Chris A. Mayhew, Karl Unterkofler

**Affiliations:** 1 Institute for Breath Research, Leopold-Franzens-Universität, Innrain 66, A-6020 Innsbruck, Austria; pawel.mochalski@uibk.ac.at (P.M.); ki.julian.ng@gmail.com (J.K.); christopher.mayhew@uibk.ac.at (C.A.M.); 2 Institute of Chemistry, Jan Kochanowski University, 25-369 Kielce, Poland; 3 Tiroler Krebsforschungsinstitut (TKFI), Innrain 66, A-6020 Innsbruck, Austria; 4 Research Center BI, University of Applied Sciences Vorarlberg, Hochschulstraße 1, A-6850 Dornbirn, Austria

**Keywords:** volatilome, VOCs, breath, end-tidal air, blood, fat, partition coefficients, modelling

## Abstract

Researchers looking for biomarkers from different sources, such as breath, urine, or blood, frequently search for specific patterns of volatile organic compounds (VOCs), often using pattern recognition or machine learning techniques. However, they are not generally aware that these patterns change depending on the source they use. Therefore, we have created a simple model to demonstrate that the distribution patterns of VOCs in fat, mixed venous blood, alveolar air, and end-tidal breath are different. Our approach follows well-established models for the description of dynamic real-time breath concentration profiles. We start with a uniform distribution of end-tidal concentrations of selected VOCs and calculate the corresponding target concentrations. For this, we only need partition coefficients, mass balance, and the assumption of an equilibrium state, which avoids the need to know the volatiles’ metabolic rates and production rates within the different compartments.

## 1. Introduction

Over the last decade, human volatilomics have been the subject of in-depth biomarker discovery studies aimed at the identification of novel biomarkers for medical diagnosis and therapy monitoring [1]. The term volatilome is commonly understood as a subset of the metabolome comprising volatile organic compounds (VOCs) within the human body. Human VOCs can be the end- or by-products of metabolic and biochemical processes occurring in the body, or originate from exogenous sources entering the human body via ingestion, inhalation, or dermal routes. Overall, the volatilome is believed to create specific biochemical signatures that contain information on the metabolic status of the organism. These chemical signatures exhibit distinct and immediate changes when diverse abnormal processes such as oxidative stress, changes in enzyme activity, carbohydrate-metabolism, lipid metabolism, modifications of proteins, or activation of genes occur and modify the body’s biochemistry.

The unique feature of the volatilomic approach is that the information on the processes in the human organism is obtained non-invasively via the analysis of volatiles emitted or secreted by the human body into its surrounding environment. These embrace, i.e., breath, skin emanations, urine, saliva, faeces, or sweat. A fundamental assumption of the volatilomic approach is that the levels of the volatile metabolites in the body excretions under study correlate with their levels in blood, and next with their levels in the tissue or organ of interest. In other words, volatilomics assumes that the volatile signatures in different excretions contain reliable information on the physiological processes occurring even in distant compartments of the organism. In this context, an in-depth understanding of the different processes governing the distribution of VOCs between different tissues, bodily fluids, and breath is of key importance for the potential of volatilomics to be fully exploited. Breath holds, in this context, a distinguished status, as it can be obtained non-invasively, rapidly, and as often as deemed without discomfort for the subject. Moreover, exhaled breath can be measured in real-time with breath-by-breath resolution using simple and cheap analyzers. All of these features render breath gas an optimal reservoir of human VOCs. Therefore, exhaled breath has become the main excretion targeted by volatilomics.

The volatilomic signatures related to a particular disease state may differ considerably depending on the bodily sample being targeted, because VOCs showing similar levels in one bodily sample can exhibit disparate concentrations in another. This stems from different physico-chemical features of VOCs affecting key parameters governing the behavior of volatiles in the human organism, such as the partition coefficients associated with blood:air $\left(\lambda_{b:\mathrm{air}}\right)$, water:air ($\lambda_{w:\mathrm{air}}$), and tissue:blood $\left(\lambda_{\mathrm{tis}:b}\right)$. This fact has important consequences for applying volatilomics: the biochemical signatures associated with a particular process occurring in the body are matrix-dependent.

Within this context, an in-depth understanding of the different processes governing the distribution and transport of VOCs into, or from different tissues, bodily fluids and bodily excretions is of key importance for the volatilomics to be fully exploited. The main goal of this paper is to demonstrate how the end-tidal breath. VOC signatures differ from those in different body regions using a modeling-based approach. For this purpose, alveolar air, blood and fat tissue have been selected. The latter was chosen due to the availability of the experimentally determined values of the fat:air partition coefficient ($\lambda_{\mathrm{fat}:\mathrm{air}}$). A particular focus is on alveolar air as it is still commonly and incorrectly equated to end-tidal air.

Blood flow (cardiac output ${\dot{Q}}_{c}$) and breath flow (alveolar ventilation ${\dot{V}}_{A}$) have a great influence on end-tidal breath concentrations. Using isoprene as a prototype of a VOC with a low blood:air partition coefficient ($\lambda_{b:\mathrm{air}}\approx 1$), King et al. [2] developed a three-compartment model (one lung and two body compartments: alveolar compartment (gas exchange), peripheral tissue containing the working muscles (with metabolism and production), and richly perfused tissue (containing the liver with metabolism and production) that dynamically describes the real-time isoprene breath concentration profile for various conditions, e.g., exercise, hyperventilation, and change of position [3], and sleep [4]. A simpler two-compartment model for VOCs with low blood:air partition coefficients was presented by Unterkofler et al. [5]. This provides total production and metabolic rates of the whole body when at rest. The model also demonstrates how to take account of inhaled concentrations, as confirmed by experiments with the inhalation of deuterated isoprene. Using acetone as a prototype of a VOC with a high blood:air partition coefficient, King et al. [6] created a four-compartment model (two lung and two body compartments: the body is divided into four distinct functional units: bronchial/mucosal compartment (gas exchange), alveolar compartment (gas exchange), liver (metabolism and production) and tissue (storage)) which describes dynamically the real-time acetone breath concentration profile for various conditions, e.g., exercise, hyperventilation, sleep [4], and rebreathing [7]. Acetone is highly water soluble, and hence it has a very high blood:air partition coefficient ($\lambda_{b:\mathrm{air}}\approx 340$), meaning that it will interact and equilibrate with the mucus surface. Therefore, the end-tidal acetone breath concentration is the bronchial concentration, which is much lower than the alveolar concentration. For this case, too, a simpler three-compartment model for VOCs with high blood:air partition coefficients was presented in Ager et al. [8], which also provides total production and metabolic rates of the whole body when at rest. The model also demonstrates how any inhaled concentrations can be taken into account.

## 2. Methods

### 2.1. Modelling VOC Concentrations in Different Body Regions

We create an example model which demonstrates how the exhaled end-tidal VOC patterns differ from the VOC patterns in different body regions. To keep the example as simple as possible, we choose two body compartments only (we also assume that the VOCs are inert (no chemical binding in blood) and that no production occurs in mouth or nose):

(i) a fat compartment with concentration $C_{\mathrm{fat}}$ (with no production and no metabolism of the VOC under consideration in the fat compartment);
(ii) a residual body compartment containing all the rest of the body with concentration $C_{\mathrm{res}}$ (with possible production and metabolism).

Further, we distinguish two cases:

Case 1: if the blood:air partition coefficient $\lambda_{b:\mathrm{air}}<10$, we use a single lung compartment with alveolar concentration $C_{A}$, as in this case the upper airways in general do not have any effect on the exhaled concentration. Then, the end-tidal concentration equals the alveolar concentration

(1) $$C_{\mathrm{end}-\mathrm{tidal}}=C_{A}.$$

Case 2: if $\lambda_{b:\mathrm{air}}>10$, we use a two compartment lung consisting of a bronchial compartment with concentration $C_{\mathrm{bro}}$ and an alveolar compartment with concentration $C_{A}$ where the two lung compartments interact by diffusion which is modelled by a conductance parameter *D*, (L/min) (see Equations (12) and (13)). It is well known that, for highly water soluble VOCs, the exhaled concentration does not equal the alveolar concentration (see e.g., [6,9]). In this case, we have

(2) $$C_{\mathrm{end}-\mathrm{tidal}}=C_{\mathrm{bro}}.$$

For both cases, the mixed venous blood concentration $C_{\bar{v}}$ is given by

(3) $$C_{\bar{v}}=q_{\mathrm{fat}}\lambda_{b:\mathrm{fat}}C_{\mathrm{fat}}+\left(1-q_{\mathrm{fat}}\right)\lambda_{b:\mathrm{res}}C_{\mathrm{res}}$$

where $q_{\mathrm{fat}}\approx 0.1$ is the relative fractional blood flow of the fat compartment. The concentration in fat (when in the state of an equilibrium and no production and no metabolism in the fat compartment occurs) can be obtained from

(4) $$C_{a}=\lambda_{b:\mathrm{fat}}C_{\mathrm{fat}}$$

where $C_{a}$ is the arterial concentration. Additionally, the arterial concentration $C_{a}$ is in equilibrium with the alveolar concentration

(5) $$C_{a}=\lambda_{b:\mathrm{air}}C_{A}.$$

In case 2, we distinguish two subcases:

(2a) if $\lambda_{b:\mathrm{air}}>100$ (and assuming no hyperventilation) we set D=0, which simplifies the formulae considerably;
(2b) D≠0 if $10<\lambda_{b:\mathrm{air}}<100$ or when hyperventilating.

### 2.2. Derivation of the Formulae for Case 1

For case 1, the model consists of three compartments. The general derivation of the compartment equations follows in analogy to the isoprene model developed by King et al. [3].

**Figure 1:**
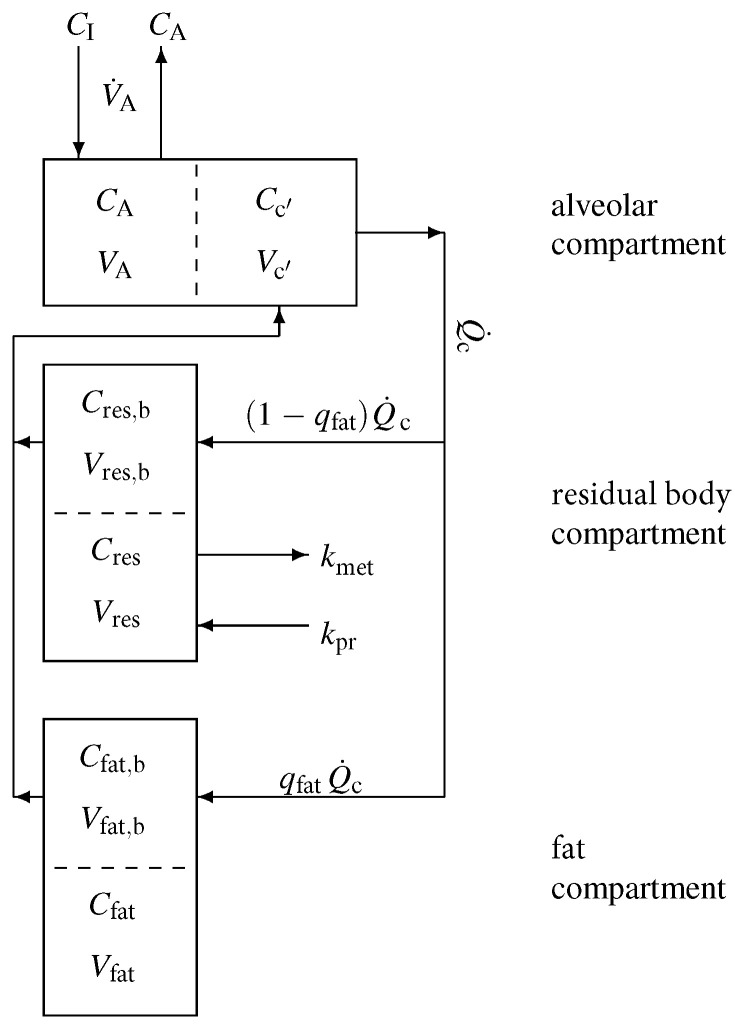
Sketch of the model structure. The body is divided into three distinct functional units: alveolar/end-capillary compartment (gas exchange), residual body compartment (metabolism and production), and fat compartment (storage). Dashed boundaries indicate a diffusion equilibrium. Here, *C* denotes the corresponding concentrations, *V* the volumes, $q_{\mathrm{fat}}$ the relative blood flow in fat, ${\dot{Q}}_{c}$ the cardiac output, ${\dot{V}}_{A}$ the alveolar ventilation, and $k_{\mathrm{pr}}$, $k_{\mathrm{met}}$ the production and metabolic rates.

A sketch of the model structure is given in Figure 1 and is described in the following. Model equations are derived by taking into account standard conservation of mass laws for the individual compartments. In view of the diffusion equilibria, the compartment capacities are governed by the *effective* volumes ${\tilde{V}}_{A}:=V_{A}+V_{c^{\prime }}\lambda_{b:\mathrm{air}}$, ${\tilde{V}}_{\mathrm{res}}:=V_{\mathrm{res}}+V_{\mathrm{res},b}\lambda_{b:\mathrm{res}}$ as well as ${\tilde{V}}_{\mathrm{fat}}:=V_{\mathrm{fat}}+V_{\mathrm{fat},b}\lambda_{b:\mathrm{fat}}$. In Appendix A, we give a brief introduction to effective volumes using the fat compartment as an example.

According to Figure 1, the mass balance equation for the alveolar compartment is

(6) $${\tilde{V}}_{A}\frac{dC_{A}}{dt}={\dot{V}}_{A}\left(C_{I}-C_{A}\right)+{\dot{Q}}_{c}\left(C_{\bar{v}}-C_{a}\right),$$

with $C_{I}$ denoting the inhaled (ambient) VOC concentration, while for the residual body and fat compartment we find that

(7) $${\tilde{V}}_{\mathrm{res}}\frac{dC_{\mathrm{res}}}{dt}=\left(1-q_{\mathrm{fat}}\right){\dot{Q}}_{c}\left(C_{a}-\lambda_{b:\mathrm{res}}C_{\mathrm{res}}\right)+k_{\mathrm{pr}}-k_{\mathrm{met}}\lambda_{b:\mathrm{res}}C_{\mathrm{res}},$$

and

(8) $${\tilde{V}}_{\mathrm{fat}}\frac{dC_{\mathrm{fat}}}{dt}=q_{\mathrm{fat}}{\dot{Q}}_{c}\left(C_{a}-\lambda_{b:\mathrm{fat}}C_{\mathrm{fat}}\right),$$

respectively. Here, the associated concentrations in mixed venous and arterial blood are given by

(9) $$C_{\bar{v}}:=q_{\mathrm{fat}}\lambda_{b:\mathrm{fat}}C_{\mathrm{fat}}+\left(1-q_{\mathrm{fat}}\right)\lambda_{b:\mathrm{res}}C_{\mathrm{res}}$$

and Equation (5), respectively.

In an equilibrium state at rest, all derivatives on the left side of the three differential equations are zero and hence we face three linear algebraic equations to solve.

Equation (6) yields the classical Farhi equation when $C_{I}=0$ (compare with, e.g., Equation (3) in [5], $C_{\mathrm{end}-\mathrm{tidal}}=C_{A}$)

(10) $$C_{A}=\frac{C_{\bar{v}}}{\lambda_{b:\mathrm{air}}+\frac{{\dot{V}}_{A}}{{\dot{Q}}_{c}}}=\frac{q_{\mathrm{fat}}\lambda_{b:\mathrm{fat}}C_{\mathrm{fat}}+\left(1-q_{\mathrm{fat}}\right)\lambda_{b:\mathrm{res}}C_{\mathrm{res}}}{\lambda_{b:\mathrm{air}}+\frac{{\dot{V}}_{A}}{{\dot{Q}}_{c}}}.$$

Equation (8) yields $C_{a}=\lambda_{b:\mathrm{fat}}C_{\mathrm{fat}}$ and, finally, using $\lambda_{b:\mathrm{fat}}=\frac{\lambda_{b:\mathrm{air}}}{\lambda_{\mathrm{fat}:\mathrm{air}}}$

(11) $$C_{A}=\frac{\lambda_{b:\mathrm{fat}}}{\lambda_{b:\mathrm{air}}}\;C_{\mathrm{fat}}=\frac{1}{\lambda_{\mathrm{fat}:\mathrm{air}}}\;C_{\mathrm{fat}}.$$

It should be noted that, as we do not know the production and metabolic rates, $k_{\mathrm{pr}}$ and $k_{\mathrm{met}}$, we will not use Equation (7) going forward. However, given $C_{\mathrm{end}-\mathrm{tidal}}$, we can calculate $C_{a}$ by using Equation (5) and then $C_{\mathrm{fat}}$ from Equation (4). $C_{\bar{v}}$ is determined by Equation (10) and furthermore $\lambda_{b:\mathrm{res}}C_{\mathrm{res}}$ follows.

### 2.3. Derivation of the Formulae for Case 2, $\lambda_{b:\mathrm{air}}>10$

For this case, we adopt the four compartment model developed for acetone by King et al. [6]. We use the same model structure, but instead of the tissue compartment we use a fat compartment, and instead of the liver compartment we use a residual body compartment.

**Figure 2:**
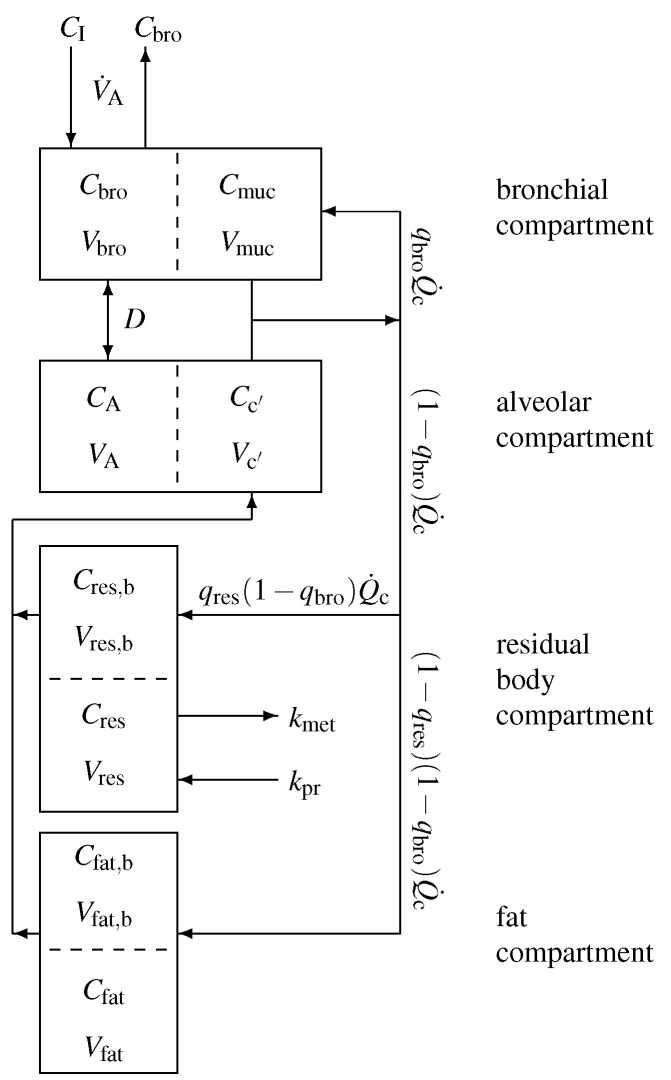
Sketch of the model structure. The body is divided into four distinct functional units: bronchial/mucosal compartment (gas exchange), alveolar/end-capillary compartment (gas exchange), residual body (metabolism and production), and fat (storage). Dashed boundaries indicate a diffusion equilibrium. The conductance parameter *D* has units of volume divided by time and quantifies an effective diffusion barrier between the bronchial and the alveolar tract. Here, *C* denotes the corresponding concentrations, *V* the volumes, $q_{\mathrm{res}},q_{\mathrm{bro}}$ the relative corresponding blood flows, ${\dot{Q}}_{c}$ the cardiac output, ${\dot{V}}_{A}$ the alveolar ventilation, and $k_{\mathrm{pr}}$, $k_{\mathrm{met}}$ the production and metabolic rates.

In order to capture the gas exchange and tissue distribution mechanisms, the model consists of four different compartments. A sketch of the model structure is given in Figure 2 and is described in the following.

Model equations are derived by taking into account standard conservation of mass laws for the individual compartments. Local diffusion equilibria are assumed to hold at the air-tissue, tissue-blood, and air-blood interfaces, the ratio of the corresponding concentrations being described by the appropriate partition coefficients, e.g., $\lambda_{b:\mathrm{air}}$. Unlike for low blood soluble compounds, the amount of highly soluble gas dissolved in the local blood volume of perfused compartments cannot generally be neglected, as it might significantly increase the corresponding capacities. This is particularly true for the airspace compartments. As reliable data for some local blood volumes could not be found, in order not to overload the model with too many hypothetical parameters, we will use the effective compartment volumes ${\tilde{V}}_{\mathrm{bro}}:=V_{\mathrm{bro}}+V_{\mathrm{muc}}\lambda_{\mathrm{muc}:\mathrm{air}}$, ${\tilde{V}}_{A}:=V_{A}+V_{c^{\prime }}\lambda_{b:\mathrm{air}}$, ${\tilde{V}}_{\mathrm{res}}:=V_{\mathrm{res}}+V_{\mathrm{res},b}\lambda_{b:\mathrm{res}}$, as well as ${\tilde{V}}_{\mathrm{fat}}:=V_{\mathrm{fat}}$, and neglect blood volumes for the mucosal and tissue compartment.

It should be noted that, though the volumes $V_{\mathrm{bro}}$ and $V_{\mathrm{muc}}$, as well as the relative bronchial blood flow $q_{\mathrm{bro}}$, are small, the effective volume ${\tilde{V}}_{\mathrm{bro}}$ is large as $\lambda_{\mathrm{muc}:\mathrm{air}}$ is large.

According to Figure 2, for the bronchial compartment we find that

(12) $$\frac{dC_{\mathrm{bro}}}{dt}{\tilde{V}}_{\mathrm{bro}}={\dot{V}}_{A}\left(C_{I}-C_{\mathrm{bro}}\right)+D\left(C_{A}-C_{\mathrm{bro}}\right)+q_{\mathrm{bro}}{\dot{Q}}_{c}\left(C_{a}-\frac{\lambda_{\mathrm{muc}:\mathrm{air}}}{\lambda_{\mathrm{muc}:b}}C_{\mathrm{bro}}\right)$$

with $C_{I}$ denoting the inhaled (ambient) VOC concentration, while the mass balance equations for the alveolar, residual body, and fat compartment read

(13) $$\frac{dC_{A}}{dt}{\tilde{V}}_{A}=D\left(C_{\mathrm{bro}}-C_{A}\right)+\left(1-q_{\mathrm{bro}}\right){\dot{Q}}_{c}\left(C_{\bar{v}}-\lambda_{b:\mathrm{air}}C_{A}\right),$$

(14) $$\frac{dC_{\mathrm{res}}}{dt}{\tilde{V}}_{\mathrm{res}}=k_{\mathrm{pr}}-k_{\mathrm{met}}\lambda_{b:\mathrm{res}}C_{\mathrm{res}}+\left(1-q_{\mathrm{fat}}\right)\left(1-q_{\mathrm{bro}}\right){\dot{Q}}_{c}\left(C_{a}-\lambda_{b:\mathrm{res}}C_{\mathrm{res}}\right),$$

and

(15) $$\frac{dC_{\mathrm{fat}}}{dt}{\tilde{V}}_{\mathrm{fat}}=q_{\mathrm{fat}}\left(1-q_{\mathrm{bro}}\right){\dot{Q}}_{c}\left(C_{a}-\lambda_{b:\mathrm{fat}}C_{\mathrm{fat}}\right),$$

respectively. Here,

(16) $$C_{\bar{v}}:=\left(1-q_{\mathrm{fat}}\right)\lambda_{b:\mathrm{res}}C_{\mathrm{res}}+q_{\mathrm{fat}}\lambda_{b:\mathrm{fat}}C_{\mathrm{fat}}$$

and

(17) $$C_{a}:=\left(1-q_{\mathrm{bro}}\right)\lambda_{b:\mathrm{air}}C_{A}+q_{\mathrm{bro}}\frac{\lambda_{\mathrm{muc}:\mathrm{air}}}{\lambda_{\mathrm{muc}:b}}\;C_{\mathrm{bro}}$$

are the concentrations in mixed venous and arterial blood, respectively. Moreover, the measured end-tidal breath concentrations equals the bronchial levels, i.e.,

(18) $$C_{\mathrm{measured}}=C_{\mathrm{bro}}.$$

In an equilibrium state at rest, all derivatives at the left side of the four differential equations are zero and hence we find four linear algebraic equations to solve (The completely decoupled case $D=q_{\mathrm{bro}}=0$ will be excluded as we continue, as it lacks physiological relevance).

Equation (15) yields

(19) $$C_{a}=\lambda_{b:\mathrm{fat}}C_{\mathrm{fat}}$$

again, and hence

(20) $$C_{\mathrm{fat}}=\frac{1}{\lambda_{b:\mathrm{fat}}}\;C_{a}=\frac{1}{\lambda_{b:\mathrm{fat}}}\left(\left(1-q_{\mathrm{bro}}\right)\lambda_{b:\mathrm{air}}C_{A}+q_{\mathrm{bro}}\frac{\lambda_{\mathrm{muc}:\mathrm{air}}}{\lambda_{\mathrm{muc}:b}}\;C_{\mathrm{bro}}\right).$$

As we do not know the production and metabolic rate ($k_{\mathrm{pr}}$ and $k_{\mathrm{met}}$, respectively), we will not use Equation (14) henceforth.

#### 2.3.1. Case 2a: $\lambda_{b:\mathrm{air}}>100$

If $\lambda_{b:\mathrm{air}}>100$ we can ignore the influence of diffusion, i.e., we set D=0, when not hyperventilating. The end-tidal concentration equals the bronchial concentration $C_{\mathrm{bro}}$, i.e., $C_{\mathrm{end}-\mathrm{tidal}}=C_{\mathrm{bro}}$ and we find an analogy to Equation (21) in [6] (when the inhaled concentration $C_{I}=0$)

(21) $$C_{\mathrm{bro}}=\frac{\left(1-q_{\mathrm{bro}}\right)\lambda_{b:\mathrm{air}}C_{A}}{\left(1-q_{\mathrm{bro}}\right)\frac{\lambda_{\mathrm{muc}:\mathrm{air}}}{\lambda_{\mathrm{muc}:b}}+\frac{{\dot{V}}_{A}}{q_{\mathrm{bro}}{\dot{Q}}_{c}}}=\frac{\left(1-q_{\mathrm{bro}}\right)C_{\bar{v}}}{\left(1-q_{\mathrm{bro}}\right)\frac{\lambda_{\mathrm{muc}:\mathrm{air}}}{\lambda_{\mathrm{muc}:b}}+\frac{{\dot{V}}_{A}}{q_{\mathrm{bro}}{\dot{Q}}_{c}}}=\frac{C_{a}}{\frac{\lambda_{\mathrm{muc}:\mathrm{air}}}{\lambda_{\mathrm{muc}:b}}+\frac{{\dot{V}}_{A}}{q_{\mathrm{bro}}{\dot{Q}}_{c}}},$$

corresponding to purely bronchial gas exchange.

Given $C_{\mathrm{end}-\mathrm{tidal}}$, we can calculate $C_{A}$, $C_{\bar{v}}$, and $C_{a}$ using Equation (21) and then $C_{\mathrm{fat}}$ using Equation (19). Furthermore, $C_{\bar{v}}$ determines $\lambda_{b:\mathrm{res}}C_{\mathrm{res}}$ according to Equation (16).

In addition we see from Equation (21) that

(22) $$\lambda_{b:\mathrm{air}}C_{A}=C_{\bar{v}}\approx C_{a}=\lambda_{b:\mathrm{fat}}C_{\mathrm{fat}}\;\mathrm{and}\;\mathrm{hence}\;\lambda_{b:\mathrm{res}}C_{\mathrm{res}}\approx \lambda_{b:\mathrm{fat}}C_{\mathrm{fat}}$$

as $q_{\mathrm{bro}}$ is very small. The contribution from the fat compartment is small, too, as $q_{\mathrm{fat}}=0.1$.

#### 2.3.2. Case 2b: D≠0 or $10<\lambda_{b:\mathrm{air}}<100$

Solving the algebraic equations in this case yields ($C_{\mathrm{end}-\mathrm{tidal}}=C_{\mathrm{bro}}$)

(23) $$C_{\mathrm{bro}}=\frac{C_{\bar{v}}\left(\left(1-q_{\mathrm{bro}}\right)+\frac{D}{\lambda_{b:\mathrm{air}}q_{\mathrm{bro}}{\dot{Q}}_{c}}\right)}{\left(1-q_{\mathrm{bro}}\right)\frac{\lambda_{\mathrm{muc}:\mathrm{air}}}{\lambda_{\mathrm{muc}:b}}+\frac{{\dot{V}}_{A}}{q_{\mathrm{bro}}{\dot{Q}}_{c}}+\frac{D}{\left(1-q_{\mathrm{bro}}\right){\dot{Q}}_{c}}\left(\frac{{\left(1-q_{\mathrm{bro}}\right)}^{2}}{q_{\mathrm{bro}}}+\frac{\lambda_{\mathrm{muc}:\mathrm{air}}\left(1-q_{\mathrm{bro}}\right)}{\lambda_{\mathrm{muc}:b}\lambda_{b:\mathrm{air}}}+\frac{{\dot{V}}_{A}}{\lambda_{b:\mathrm{air}}q_{\mathrm{bro}}{\dot{Q}}_{c}}\right)},$$

(24) $$=\frac{C_{a}\left(1+\frac{D}{\lambda_{b:\mathrm{air}}q_{\mathrm{bro}}\left(1-q_{\mathrm{bro}}\right){\dot{Q}}_{c}}\right)}{\frac{\lambda_{\mathrm{muc}:\mathrm{air}}}{\lambda_{\mathrm{muc}:b}}+\frac{{\dot{V}}_{A}}{q_{\mathrm{bro}}{\dot{Q}}_{c}}+\frac{D}{q_{\mathrm{bro}}{\dot{Q}}_{c}}\left(1+\frac{q_{\mathrm{bro}}\lambda_{\mathrm{muc}:\mathrm{air}}{\dot{Q}}_{c}}{\left(1-q_{\mathrm{bro}}\right)\lambda_{\mathrm{muc}:b}\lambda_{b:\mathrm{air}}}\right)},$$

(25) $$=\frac{C_{A}\;\left(\lambda_{b:\mathrm{air}}\left(1-q_{\mathrm{bro}}\right)+\frac{D}{q_{\mathrm{bro}}{\dot{Q}}_{c}}\right)}{\frac{\lambda_{\mathrm{muc}:\mathrm{air}}}{\lambda_{\mathrm{muc}:b}}\left(1-q_{\mathrm{bro}}\right)+\frac{{\dot{V}}_{A}}{q_{\mathrm{bro}}{\dot{Q}}_{c}}+\frac{D}{q_{\mathrm{bro}}{\dot{Q}}_{c}}},$$

(26) $$=\frac{\lambda_{b:\mathrm{fat}}C_{\mathrm{fat}}\left(1+\frac{D}{\lambda_{b:\mathrm{air}}q_{\mathrm{bro}}\left(1-q_{\mathrm{bro}}\right){\dot{Q}}_{c}}\right)}{\frac{\lambda_{\mathrm{muc}:\mathrm{air}}}{\lambda_{\mathrm{muc}:b}}+\frac{{\dot{V}}_{A}}{q_{\mathrm{bro}}{\dot{Q}}_{c}}+\frac{D}{q_{\mathrm{bro}}{\dot{Q}}_{c}}\left(1+\frac{q_{\mathrm{bro}}\lambda_{\mathrm{muc}:\mathrm{air}}{\dot{Q}}_{c}}{\left(1-q_{\mathrm{bro}}\right)\lambda_{\mathrm{muc}:b}\lambda_{b:\mathrm{air}}}\right)}.$$

Taking the limit D→0 in these formulae recovers the case 2a, and taking the limit $q_{\mathrm{bro}}\to 0$ and D→∞ in these formulae recovers case 1.

The diffusion constant *D* depends on $\lambda_{b:\mathrm{air}}$ and tends to zero when $\lambda_{b:\mathrm{air}}\to \infty$ and becomes infinite when $\lambda_{b:\mathrm{air}}\to 0$. Hence, we model it by use of exponential functions:

$$D\left(\lambda_{b:\mathrm{air}}\right)=\frac{a\;e^{-c\;\lambda_{b:\mathrm{air}}}}{1-e^{-d\;\lambda_{b:\mathrm{air}}}}\;\approx \;a\;e^{-c\;\lambda_{b:\mathrm{air}}}\;\mathrm{for}\;\lambda_{b:\mathrm{air}}>10,\;a,c,d>0.$$

For $D(\lambda_{b:\mathrm{air}})$ at rest in the range $10<\lambda_{b:\mathrm{air}}<100$, we use the following approximation

(27) $$D\left(\lambda_{b:\mathrm{air}}\right)=215\;e^{-0.075\;\lambda_{b:\mathrm{air}}}.$$

### 2.4. Nominal Data for Modelling

We use the following nominal values for cardiac output, alveolar ventilation, and relative blood flows at rest: ${\dot{Q}}_{c}=5$ L/min, ${\dot{V}}_{A}=5.2$ L/min [10], $q_{\mathrm{bro}}=0.01$ [11], and $q_{\mathrm{fat}}=0.1$.

For the conversion from ppb to nmol/L, we use the molar volume at sea level ($p_{0}$ = 101,325 Pascal) and the end-tidal temperature of 32 °C, which yields $V_{mol}=25.04$ L.

The decrease in solubility in the mucosa of highly soluble VOCs, such as acetone, (expressed as the water:air partition coefficient $\lambda_{\mathrm{muc}:\mathrm{air}}$) with increasing temperature can be described in the ambient temperature range by a Van ’t Hoff-type equation (Staudinger et al. [12]):

(28) $$\log_{10}\lambda_{\mathrm{muc}:\mathrm{air}}\left(T\right)=-A+\frac{B}{T+273.15}.$$

The blood:air partition coefficient $\lambda_{b:\mathrm{air}}$ will always refer to 37 °C. Similarly, the partition coefficient between mucosa and blood is treated as a constant defined by

(29) $$\lambda_{\mathrm{muc}:b}:=\lambda_{\mathrm{muc}:\mathrm{air}}\left(37\;{}^{\circ}C\right)/\lambda_{b:\mathrm{air}}.$$

Note that if the airway temperature is below 37 °C, we always have that $\lambda_{\mathrm{muc}:\mathrm{air}}/\lambda_{\mathrm{muc}:b}$$\geq \lambda_{b:\mathrm{air}},$ as $\lambda_{\mathrm{muc}:\mathrm{air}}$ is monotonically decreasing with increasing temperature. The factor $\frac{\lambda_{\mathrm{muc}:\mathrm{air}}}{\lambda_{\mathrm{muc}:b}}$ in our formulae hence equals $\frac{\lambda_{\mathrm{muc}:\mathrm{air}}}{\lambda_{\mathrm{muc}:b}}=\frac{\lambda_{\mathrm{muc}:\mathrm{air}}\left(32\;{}^{\circ}C\right)}{\lambda_{\mathrm{muc}:\mathrm{air}}\left(37\;{}^{\circ}C\right)}\;\lambda_{b:\mathrm{air}}$.

A compilation of various partition coefficients can be found in the report of Sander [13].

## 3. Results

To illustrate how a particular VOC profile changes during the transfer of VOCs between different tissues, fluids, and excretions, 16 volatiles have been selected, namely; n-pentane, n-hexane, isoprene, benzene, n-nonane, ethylbenzene, p-xylene, DL-limonene, styrene, 1,2,3-trimethylbenzene, ethyl acetate, methyl acetate, 2-pentanone, acetone, 2-propanol, and ethanol. Although the main selection criterion was the availability of the experimentally determined values of blood:air and blood:fat partition coefficients, an effort was made to include species exhibiting a wide range of blood:air partition coefficient values. Effectively, the blood:air partition coefficients of the preselected VOCs cover more than three orders of magnitude and range from 0.42–1500. The preselected species, together with their key parameters used in this paper, are listed in Table 1.

The set of compounds under study embraces very lipophilic species (n-pentane, n-hexene, or isoprene) and hydrophilic compounds (ethanol, 2-propanol, or acetone) as well as representants of all cases discussed in the method section. Moreover, an end-tidal pattern of VOCs under study at equal concentrations of 4 nmol × L^−1^ at 32 °C and 1 bar (100 ppb) has been put forward as a starting point for the demonstration of the above-mentioned discrepancies. In breath analysis, the end-tidal concentration is defined as the average concentration of a VOC in the last phase of exhalation. Although such an end-tidal signature of 100 ppb is chosen just for demonstration, it allows us to show in a very illustrative way the discussed effects. 

### 3.1. Alveolar Air versus End-Tidal Air

Although a number of analytical techniques can be used to detect and track VOCs in human breath, they predominantly provide end-tidal breath concentrations of breath volatiles $C_{\mathrm{end}-\mathrm{tidal}}=C_{\mathrm{measured}}$. In the classical approach, the end-tidal air is equated to the alveolar air, and the corresponding arterial concentrations can be assessed by simply multiplying this value by the blood:air partition coefficient $\lambda_{b:\mathrm{air}}$ at body temperature. This approach stems from the classical Farhi description of the pulmonary inert gas exchange [14]. However, the Fahri model fails to describe the exhalation kinetics of highly soluble trace gases [6,9,15]. This class of compounds has been demonstrated to significantly interact with the water-like mucus membrane lining the conductive airways, an effect which has come to be known as the wash-in/wash-out phenomenon [15]. As a consequence, breath concentrations of hydrophilic volatiles tend to be decreased on their way up from the alveoli via the respiratory tract to the airway opening. The resulting discrepancies between the alveolar air and the measured end-tidal air can be significant and depend on a number of factors such as airway temperature profiles, airway perfusion, breathing patterns, and primarily the VOC’s blood:air partition coefficient.

The blood:air partition coefficient is a complex parameter resulting from two processes occurring in the blood, namely partitioning (solubility) and binding [16]. The former is associated with the composition of plasma such as water, lipids and phospholipids content, and erythrocytes. The binding, in turn, is determined by plasma proteins and haemoglobin. While the partitioning fraction of $\lambda_{b:\mathrm{air}}$ is not expected to change as a function of concentration, the binding process exhibits saturation and changes with the concentration of a given VOC [16]. The $\lambda_{b:\mathrm{air}}$ also depends on the specific physico-chemical features of a particular compound and can differ considerably between different species. It is worth noting that the real values of $\lambda_{b:\mathrm{air}}$ for many volatiles are unknown; however, they can be estimated using predictive approaches [17,18].

**Figure 3:**
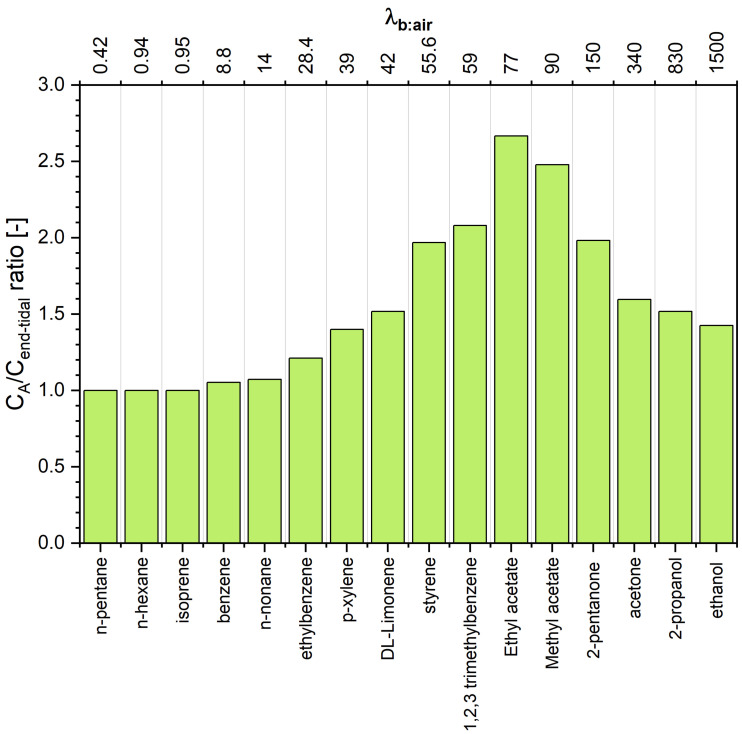
Concentration ratio between alveolar and end-tidal air $C_{A}/C_{\mathrm{end}-\mathrm{tidal}}$ for VOCs under scrutiny. Compounds are ordered with respect to increasing blood:air partition coefficient $\lambda_{b:\mathrm{air}}$.

The estimated concentrations of VOCs under scrutiny in alveolar air are presented in Table 1 and Figure 3. A scrutiny of Table 1 reveals interesting features of the end-tidal and alveolar signatures. For volatiles exhibiting low solubility in blood (defined to compounds having $\lambda_{b:\mathrm{air}}<10$) the end-tidal levels are close to the alveoli levels, which agrees with the numerous literature data [2,3,19,20]. More pronounced discrepancies can be observed for VOCs with $\lambda_{b:\mathrm{air}}$ close to 10. For instance, for benzene ($\lambda_{b:\mathrm{air}}=8.8$) the difference $C_{A}-C_{\mathrm{end}-\mathrm{tidal}}$ amounts to 5%. Thus, the assumption that $C_{A}=C_{\mathrm{end}-\mathrm{tidal}}=C_{\mathrm{measured}}$ is reasonable for compounds from this class. Of importance for breath analysis is that VOCs with low blood solubility react very sensitively to changes in ventilation and perfusion, which can be incorrectly identified as fluctuations of their blood levels [3,5]. At the other extreme are hydrophilic VOCs having $\lambda_{b:\mathrm{air}}>100$ that also exchange in the upper airways [6,7,8,21]. As a consequence, their end-tidal levels are lower than those in the alveoli, as has been explained in detail by King et al. [6]. In brief, this effect is due to an effective concentration gradient between the conducting airways and the alveolar space. Interestingly, the difference $C_{A}-C_{\mathrm{end}-\mathrm{tidal}}$ (as well as the ratio) has a maximum at $\lambda_{b:\mathrm{air}}\approx 80$, and then decreases with increasing $\lambda_{b:\mathrm{air}}$. For instance, the concentration of 2-pentanone ($\lambda_{b:\mathrm{air}}=150$) in alveolar air is almost twice as high as that in the end-tidal air, whereas, for ethanol ($\lambda_{b:\mathrm{air}}=1500$) this factor amounts to 1.4. This dependence stems from the fact that the contribution of the factor $\frac{{\dot{V}}_{A}}{q_{\mathrm{bro}}{\dot{Q}}_{c}}$ (that amounts at rest to approximately 100) to the denominator of the Equation (21) becomes less significant with increasing $\lambda_{b:\mathrm{air}}$. Thus, to obtain alveolar concentrations of VOCs exhibiting $\lambda_{b:\mathrm{air}}>100$, the measured end-tidal levels should be converted using Equation (21). This conversion requires the knowledge of several parameters $(q_{\mathrm{bro}},\;{\dot{V}}_{A},\;{\dot{Q}}_{c},\;\lambda_{\mathrm{muc}:\mathrm{air}},\;\lambda_{\mathrm{muc}:b},$ and $\lambda_{b:\mathrm{air}}$). However, Equation (21) can be further simplified. At rest, the value of $q_{\mathrm{bro}}$ amounts approximately to 0.01, and the ratio of alveolar ventilation to cardiac output equals approximately 1. Moreover, the ratio $\frac{\lambda_{\mathrm{muc}:\mathrm{air}}}{\lambda_{\mathrm{muc}:b}}$ is given by

$$\frac{\lambda_{\mathrm{muc}:\mathrm{air}}}{\lambda_{\mathrm{muc}:b}}=\frac{\lambda_{\mathrm{muc}:\mathrm{air}}\left(32\;{}^{\circ}C\right)}{\lambda_{\mathrm{muc}:\mathrm{air}}\left(37\;{}^{\circ}C\right)}\;\lambda_{b:\mathrm{air}},$$

and the factor $\frac{\lambda_{\mathrm{muc}:\mathrm{air}}\left(32\;{}^{\circ}C\right)}{\lambda_{\mathrm{muc}:\mathrm{air}}\left(37\;{}^{\circ}C\right)}$ can be estimated using the water:air partition coefficients at 32 °C and 37 °C [13]. Moreover, as the temperature dependences of the Henry solubility (described with the Van ’t Hoff equation) of VOCs under scrutiny are quite similar (see Sander [13]), the factor $\frac{\lambda_{\mathrm{muc}:\mathrm{air}}\left(32\;{}^{\circ}C\right)}{\lambda_{\mathrm{muc}:\mathrm{air}}\left(37\;{}^{\circ}C\right)}$ can further be estimated to be around 1.35. Consequently, the knowledge of only one parameter, namely the blood:air partition coefficient, is required to roughly estimate the alveolar concentrations of VOCs with $\lambda_{b:\mathrm{air}}>100$ at rest:

(30) $$C_{A}=\left(1.35+\frac{100}{\lambda_{b:\mathrm{air}}}\right)\;C_{\mathrm{end}-\mathrm{tidal}}.$$

For 2-pentanone, acetone, 2-propanol, and ethanol, the deviation from values calculated using Equation (21) is smaller than 3.5%. Moreover, for compounds with $\lambda_{b:\mathrm{air}}\gg \frac{{\dot{V}}_{A}}{q_{\mathrm{bro}}{\dot{Q}}_{c}}\approx 100$, the $\frac{{\dot{V}}_{A}}{q_{\mathrm{bro}}{\dot{Q}}_{c}}$ factor can be neglected and $C_{A}$ can be estimated using only the $\frac{\lambda_{\mathrm{muc}:\mathrm{air}}\left(32\;{}^{\circ}C\right)}{\lambda_{\mathrm{muc}:\mathrm{air}}\left(37\;{}^{\circ}C\right)}$ ratio. Thus, Equation (30) can be further simplified to

(31) $$C_{A}=\frac{\lambda_{\mathrm{muc}:\mathrm{air}}\left(32\;{}^{\circ}C\right)}{\lambda_{\mathrm{muc}:\mathrm{air}}\left(37\;{}^{\circ}C\right)}\;C_{\mathrm{end}-\mathrm{tidal}}\approx 1.35\;C_{\mathrm{end}-\mathrm{tidal}}$$

and

(32) $$C_{a}\approx C_{\bar{v}}\approx 1.35*\lambda_{b:\mathrm{air}}\;C_{\mathrm{end}-\mathrm{tidal}}.$$

**Example** **1.**  *It is well known (see M. P. Hlastala [22]) that the “blood–breath ratio" BBR $:=\frac{C_{\bar{v}}}{C_{\mathrm{end}-\mathrm{tidal}}}$ and the blood:air partition coefficient $\lambda_{b:\mathrm{air}}$ for ethanol are different. Equation (32) now yields the following correction for ethanol:*

$$\begin{array}{c} \mathrm{BBR}\approx 1.35*\lambda_{b:\mathrm{air}}. \end{array}$$

We emphasize that hyperventilation also changes the end-tidal concentration for VOCs with high blood:air partition coefficients, as shown for acetone in Figure 6 in [6]. In Table 1, we simulate the effect of hyperventilation (V_A_ = 10.4) for different values of *D* for acetone.

The last case (2b) embraces compounds with $10<\lambda_{b:\mathrm{air}}<100$. This set of compounds exhibits an interesting relation between alveolar and end-tidal breath levels. The $\frac{C_{A}}{C_{\mathrm{end}-\mathrm{tidal}}}$ ratio increases with increasing $\lambda_{b:\mathrm{air}}$ to reach a maximum of 2.7 for $\lambda_{b:\mathrm{air}}\approx 80$. For higher $\lambda_{b:\mathrm{air}}$ values it starts to decline, as in the case of VOCs from the case 2a.

We remark that the existence of a maximum of the function $\frac{C_{A}}{C_{\mathrm{end}-\mathrm{tidal}}}$ comes from the fact that the function $D(\lambda_{b:\mathrm{air}})$ is a strictly convex monotonically decreasing function. Smaller values of $q_{\mathrm{bro}}$ than 0.01 will shift the location of this maximum to a value of $\lambda_{b:\mathrm{air}}$ larger than 80.

A scrutiny of Table 1 and Figure 3 also reveals that the wash-in/wash-out mechanism particularly strongly affects the VOCs with $50<\lambda_{b:\mathrm{air}}<150$, resulting in $\frac{C_{A}}{C_{\mathrm{end}-\mathrm{tidal}}}$ ratios greater than 1.5.

### 3.2. Blood and Fat VOC Signatures

**Figure 4:**
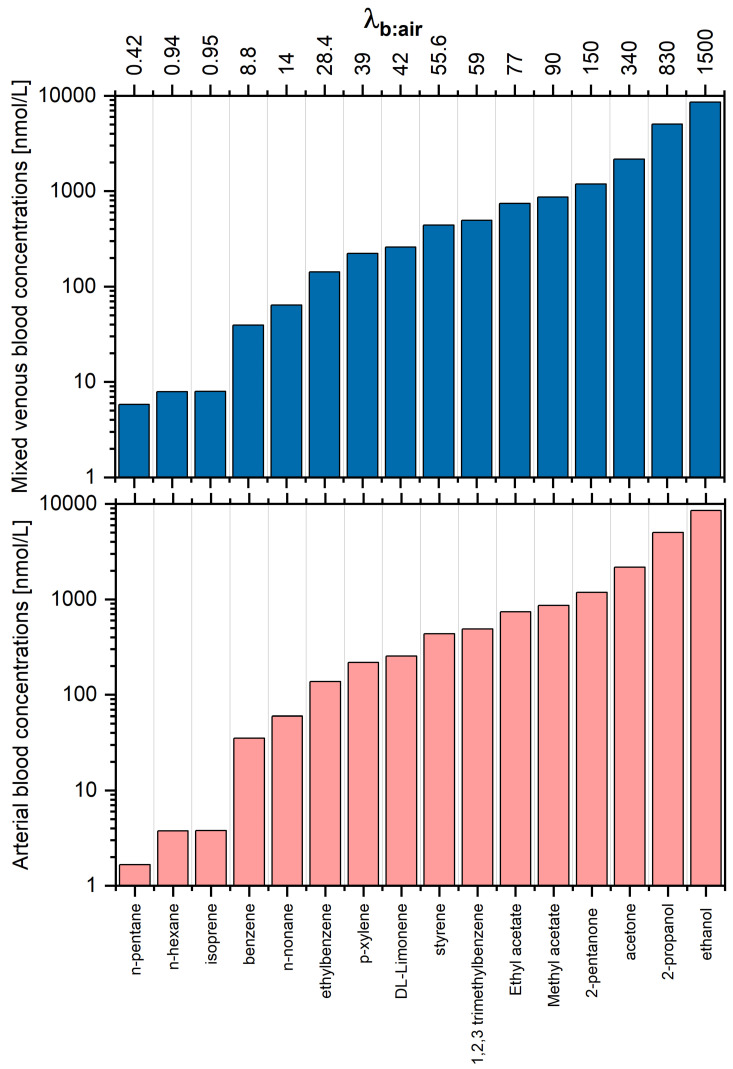
Estimated concentrations of VOCs under study in arterial and mixed venous blood, assuming that the end-tidal concentration is 4 nmol/L each. Compounds are ordered with respect to increasing blood:air partition coefficient $\lambda_{b:\mathrm{air}}$.

The estimated concentrations of VOCs under study in arterial and mixed venous blood are presented in Table 1 and Figure 4. Due to the aforementioned relations between the end-tidal breath and alveolar breath and differences in values of blood:air partition coefficients, it is not surprising that the VOC patterns in blood and end-tidal breath exhibit even more pronounced disparities. For instance, the same end-tidal levels of VOCs correspond to blood concentrations that vary by over almost four orders of magnitude. Thus, compounds with high relative abundance in blood can exhibit low abundance in breath and vice-versa. This may be a reason for the discrepancies observed between studies exploiting different biological samples such as blood and breath towards the identification of potential VOC disease markers. Comparison of VOCs in breath and blood of healthy volunteers have been investigated, e.g., in [23,24,25]. VOCs in breath and urine headspace were compared in [26]; furthermore, the urine headspace was investigated in [27].

**Figure 5:**
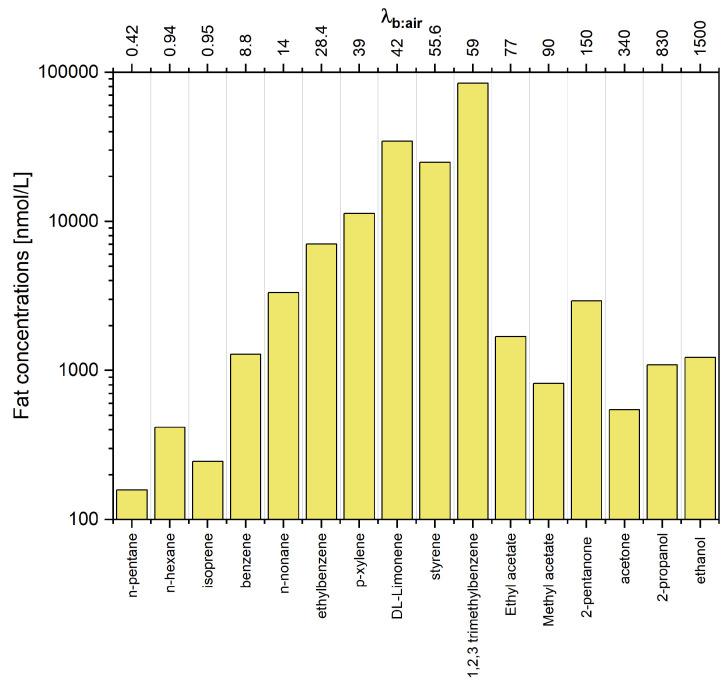
Estimated concentrations of VOCs under study in fat tissue. Compounds are ordered with respect to increasing blood:air partition coefficient $\lambda_{b:\mathrm{air}}$.

It is not surprising that the arterial and mixed venous blood VOC signature are very similar. The main difference concerns the very low water-soluble species ($\lambda_{b:\mathrm{air}}<1$) that exhibit arterial to mixed venous blood ratios of 2–3. As volatilomics usually targets mixed venous blood, the VOC profiles associated with this fluid are the subject of the biomarker discovery. A similar effect holds true for the concentrations in the fat compartment (see Table 1 and Figure 5). When we compare the levels of 1,2,3 trimethylbenzene and n-pentane, we see that these are identical in alveolar air but differ by a factor of 500 in the fat tissue. This simple example illustrates that the comparison of VOC patterns obtained from different fluids and breath cannot be investigated without a thorough knowledge of processes governing the circulation of VOCs in the human organism.

## 4. Conclusions

The aim of this article is to show that concentration patterns of VOCs differ substantially when investigating different body fluids. To achieve this, we have used a simple model which consisted of two body compartments only, a fat compartment as target and a residual body compartment. The reason for this choice was the availability of the blood:fat partition coefficients for VOCs with very different blood:air partition coefficients. If we assume a uniform distribution of the VOCs in end-tidal breath, we will see a completely different picture in the blood of the fat compartment, and vice versa. However, the calculation in the opposite direction would require the knowledge of metabolic and production rates, which are not known in most cases.

Consequently, the involvement of different bodily fluids and secretions in biomarker discovery within the volatilomics can result in the identification of different sets of biomarkers related to the same disease, as different matrices promote compounds with different physico-chemical features. For example, it could happen that some VOCs, which show up as significant biomarkers when looking at one matrix, might not even be detectable in other matrices due to limits of detection and vice versa. It also means that classification (e.g., disease/no-disease) algorithms trained on VOC data from one matrix cannot easily be transferred/generalized to other matrices.

Another limitation in the identification of biomarkers is contaminating artefacts, which are highlighted in Thorn’s review article [28].

## Figures and Tables

**Table 1:** Estimated concentrations of 16 VOCs in alveolar air ($C_{A}$), arterial blood($C_{a}$), venous blood ($C_{\bar{v}}$), and fat tissue ($C_{\mathrm{fat}}$) for the end-tidal levels of 4 nmol/L (100 ppb). Partition coefficient values can be found in Sander [13].

Compound	$\lambda_{b:\mathrm{air}}$	$\lambda_{\mathrm{fat}:\mathrm{air}}$	$\lambda_{w:\mathrm{air}}$	$\lambda_{w:\mathrm{air}}$	$C_{\mathrm{end}-\mathrm{tidal}}$	$C_{A}$	$C_{a}$	$C_{\bar{v}}$	$C_{\mathrm{fat}}$	D
Name			37 °C	32 °C	nmol/L					L/min
n-pentane	0.42	39.60	0.02	0.02	4.0	4.0	1.66	5.81	158.00	*∞*
hexane	0.94	104.00	0.01	0.01	4.0	4.0	3.75	7.91	415.34	*∞*
isoprene	0.95	61.50	0.19	0.24	4.0	4.0	3.79	7.95	245.61	*∞*
benzene	8.80	321.50	2.93	3.48	4.0	4.0	35.14	39.30	1283.95	*∞*
benzene	8.80	321.50	2.93	3.48	4.0	4.2	36.86	41.05	1348.70	111
n-nonane	13.90	831.00	0.0020	0.0028	4.0	4.3	59.66	63.85	3331.96	76
ethylbenzene	28.40	1764.00	1.71	2.21	4.0	4.8	137.63	141.82	7049.48	25
p-xylene	38.90	2020.00	2.36	2.85	4.0	5.6	217.49	221.69	11,294.00	12
DL-Limonene	42.00	5700.00	0.89	1.12	4.0	6.1	254.03	258.23	34,475.60	9
styrene	55.60	3180.00	4.54	5.67	4.0	7.9	435.55	439.74	24,910.70	3.32
1,2,3-trimethylbenzene	59.10	10,200.00	2.73	3.41	4.0	8.3	489.15	493.35	84,422.30	2.56
Acetic acid ethyl ester	76.80	176.00	67.49	87.68	4.0	9.6	737.00	741.19	1688.95	0.68
Acetic acid ethyl ester	76.80	176.00	67.49	87.68	4.0	10.7	813.80	817.99	1864.95	0
methyl ester	90.10	85.70	114.04	149.84	4.0	9.6	860.14	864.33	818.13	0.25
methyl ester	90.10	85.70	114.04	149.84	4.0	9.9	888.12	892.31	844.75	0
2-pentanone	150.00	372.00	148.25	189.95	4.0	7.9	1182.64	1186.84	2933.0	0.003
2-pentanone	150.00	372.00	148.25	189.95	4.0	7.9	1182.88	1187.07	2933.53	0
acetone	340.00	86.00	316.10	406.75	4.0	6.4	2162.56	2166.75	547.00	0
2-propanol	830.00	180.00	1278.47	1777.94	4.0	6.1	5025.01	5029.21	1089.76	0
ethanol	1500.00	215.00	2228.64	3021.70	4.0	5.7	8537.44	8541.63	1223.70	0
hyperventilating					4.0	7.6	2577.89	2586.28	652.06	0
with *V*${}_{A}$ = 10.4 L/min						7.4	2509.68	2518.07	634.80	1
acetone						6.8	2299.34	2307.73	581.60	5
						6.3	2124.60	2132.99	537.40	10

**Figure A1:**
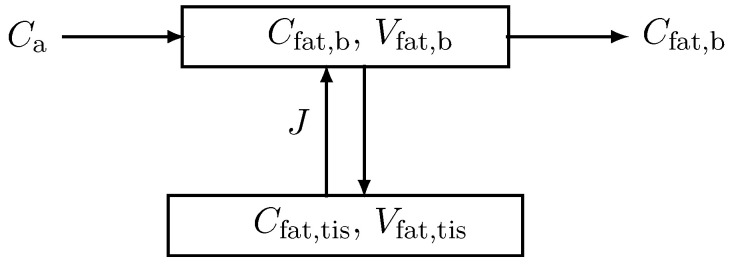
Model for the fat compartment with blood flow and tissue. Arterial blood with concentration $C_{a}$ is entering and blood with concentration $C_{\mathrm{fat},b}$ is leaving the compartment.

## Abbreviations

The following abbreviations are used in this manuscript:

Parameter	Symbol
*Compartment concentrations*	
bronchioles	$C_{\mathrm{bro}}$,$C_{\mathrm{end}-\mathrm{tidal}}$
alveoli	$C_{A}$
arterial	$C_{a}$
mixed-venous	$C_{\bar{v}}$
residual body	$C_{\mathrm{res}}$
fat	$C_{\mathrm{fat}}$
inhaled (ambient)	$C_{I}$
*Compartment volumes*	
bronchioles	$V_{\mathrm{bro}}$
mucosa	$V_{\mathrm{muc}}$
alveoli	$V_{A}$
fat	$V_{\mathrm{fat}}$
residual body	$V_{\mathrm{res}}$
*Fractional blood flows at rest*	
fractional flow bronchioles	$q_{\mathrm{bro}}$
fractional flow fat	$q_{\mathrm{fat}}$
fractional residual flow	$q_{\mathrm{res}}$
*Partition coefficients*	
blood:air	$\lambda_{b:\mathrm{air}}$
blood:residual body	$\lambda_{b:\mathrm{res}}$
blood:fat	$\lambda_{b:\mathrm{fat}}$
mucosa:air	$\lambda_{\mathrm{muc}:\mathrm{air}}$, $\lambda_{\mathrm{water}:\mathrm{air}}$
mucosa:blood	$\lambda_{\mathrm{muc}:b}$
fat:air	$\lambda_{\mathrm{fat}:\mathrm{air}}$
*Physiological parameters*	
linear metabolic rate	$k_{\mathrm{met}}$
endogenous production	$k_{\mathrm{pr}}$
cardiac output	${\dot{Q}}_{c}$
alveolar ventilation	${\dot{V}}_{A}$
conductance parameter	*D*

The end-tidal breath concentration $C_{\mathrm{end}-\mathrm{tidal}}$ is defined as $C_{\mathrm{end}-\mathrm{tidal}}:=\frac{1}{t_{2}-t_{1}}\int_{t_{1}}^{t_{2}}C\left(t\right)\;dt$, where $t_{1}$ marks the start and $t_{2}$ the end of the end-tidal phase of the capnogram.

## Appendix A

We demonstrate the concept of an effective volume ${\tilde{V}}_{\mathrm{fat}}$ by means of the effective fat compartment. The effective fat compartment consists of a blood part with concentration $C_{\mathrm{fat},b}$ and a tissue part with concentration $C_{\mathrm{fat},\mathrm{tis}}$ (in short $C_{\mathrm{fat}}$). The two parts interact by *J*.

The corresponding mass balance equation according to Figure A1 then reads

(A1) $$V_{\mathrm{fat},b}\frac{dC_{\mathrm{fat},b}}{dt}=-J+q_{\mathrm{fat}}{\dot{Q}}_{c}\left(C_{a}-C_{\mathrm{fat},b}\right)$$

and

(A2) $$V_{\mathrm{fat},\mathrm{tis}}\frac{dC_{\mathrm{fat},\mathrm{tis}}}{dt}=J$$

where $C_{a}$ denotes the arterial concentration, ${\dot{Q}}_{c}$ the cardiac output, $q_{\mathrm{fat}}$ the fractional fat blood flow, and $V_{\mathrm{fat},b},V_{\mathrm{fat},\mathrm{tis}}$ the corresponding volumes. Assuming equilibrium, we have

(A3) $$\frac{C_{\mathrm{fat},b}}{C_{\mathrm{fat},\mathrm{tis}}}=\lambda_{b:\mathrm{fat}}.$$

Eliminating $C_{\mathrm{fat},b}$ by Equation (A3), Equation (A1) becomes

(A4) $$\lambda_{b:\mathrm{fat}}V_{\mathrm{fat},b}\frac{dC_{\mathrm{fat}}}{dt}=-J+q_{\mathrm{fat}}{\dot{Q}}_{c}\left(C_{a}-\lambda_{b:\mathrm{fat}}C_{\mathrm{fat}}\right)$$

Adding Equation (A4) and (A2) yields one equation for the fat compartment with an *effective volume* ${\tilde{V}}_{\mathrm{fat}}=\lambda_{b:\mathrm{fat}}V_{\mathrm{fat},b}+V_{\mathrm{fat},\mathrm{tis}}$.

(A5) $$\underset{{\tilde{V}}_{\mathrm{fat}}}{\underset{︸}{\left(\lambda_{b:\mathrm{fat}}V_{\mathrm{fat},b}+V_{\mathrm{fat},\mathrm{tis}}\right)}}\frac{dC_{\mathrm{fat}}}{dt}=q_{\mathrm{fat}}{\dot{Q}}_{c}\left(C_{a}-\lambda_{b:\mathrm{fat}}C_{\mathrm{fat}}\right).$$

This concept can be easily extended by adding a production rate and a metabolic rate in the equation of the tissue part.
